# Accuracy of energy and nutrient intake estimation versus observed intake using 4 technology-assisted dietary assessment methods: a randomized crossover feeding study

**DOI:** 10.1016/j.ajcnut.2024.04.030

**Published:** 2024-05-06

**Authors:** Clare Whitton, Clare E Collins, Barbara A Mullan, Megan E Rollo, Satvinder S Dhaliwal, Richard Norman, Carol J Boushey, Edward J Delp, Fengqing Zhu, Tracy A McCaffrey, Sharon I Kirkpatrick, Christina M Pollard, Janelle D Healy, Amira Hassan, Shivangi Garg, Paul Atyeo, Syed Aqif Mukhtar, Deborah A Kerr

**Affiliations:** 1 Curtin School of Population Health, Curtin University, Kent Street, GPO Box U1987, Perth 6845, WA, Australia; 2 Curtin Health Innovation Research Institute, Curtin University, Kent Street, GPO Box U1987, Perth 6845, Australia; 3 School of Medical and Health Sciences, Edith Cowan University, 270 Joondalup Drive, Joondalup WA 6027, Australia; 4 School of Health Sciences, College of Health, Medicine and Wellbeing, University of Newcastle, Newcastle, Australia; 5 Food and Nutrition Research Program, Hunter Medical Research Institute, New Lambton Heights, Newcastle, Australia; 6 Enable Institute, Curtin University, Perth, Australia; 7 Obstetrics & Gynaecology Academic Clinical Program, Duke-NUS Medical School, National University of Singapore, 8 College Rd, 169857, Singapore; 8 Institute for Research in Molecular Medicine (INFORMM), Universiti Sains Malaysia, Pulau Pinang, Malaysia; 9 Singapore University of Social Sciences, 463 Clementi Road, 599494, Singapore; 10 Epidemiology Program, University of Hawaii Cancer Center, Honolulu, HI, USA; 11 School of Electrical and Computer Engineering, Purdue University, West Lafayette, IN, United States; 12 Department of Nutrition, Dietetics and Food, Monash University, Melbourne, Australia; 13 School of Public Health Sciences, University of Waterloo, Waterloo, ON, Canada; 14 Health Section, Health and Disability Branch, Australian Bureau of Statistics, Canberra, Australia

**Keywords:** Automated Self-Administered Dietary Assessment Tool, Intake24, mobile food record, interview-administered dietary recall, image-assisted, dietary assessment, validation, controlled feeding, dietary measurement error, mobile technology

## Abstract

**Background:**

Technology-assisted 24-h dietary recalls (24HRs) have been widely adopted in population nutrition surveillance. Evaluations of 24HRs inform improvements, but direct comparisons of 24HR methods for accuracy in reference to a measure of true intake are rarely undertaken in a single study population.

**Objectives:**

To compare the accuracy of energy and nutrient intake estimation of 4 technology-assisted dietary assessment methods relative to true intake across breakfast, lunch, and dinner.

**Methods:**

In a controlled feeding study with a crossover design, 152 participants [55% women; mean age 32 y, standard deviation (SD) 11; mean body mass index 26 kg/m^2^, SD 5] were randomized to 1 of 3 separate feeding days to consume breakfast, lunch, and dinner, with unobtrusive weighing of foods and beverages consumed. Participants undertook a 24HR the following day [Automated Self-Administered Dietary Assessment Tool-Australia (ASA24); Intake24-Australia; mobile Food Record-Trained Analyst (mFR-TA); or Image-Assisted Interviewer-Administered 24-hour recall (IA-24HR)]. When assigned to IA-24HR, participants referred to images captured of their meals using the mobile Food Record (mFR) app. True and estimated energy and nutrient intakes were compared, and differences among methods were assessed using linear mixed models.

**Results:**

The mean difference between true and estimated energy intake as a percentage of true intake was 5.4% (95% CI: 0.6, 10.2%) using ASA24, 1.7% (95% CI: −2.9, 6.3%) using Intake24, 1.3% (95% CI: −1.1, 3.8%) using mFR-TA, and 15.0% (95% CI: 11.6, 18.3%) using IA-24HR. The variances of estimated and true energy intakes were statistically significantly different for all methods (*P* < 0.01) except Intake24 (*P* = 0.1). Differential accuracy in nutrient estimation was present among the methods.

**Conclusions:**

Under controlled conditions, Intake24, ASA24, and mFR-TA estimated average energy and nutrient intakes with reasonable validity, but intake distributions were estimated accurately by Intake24 only (energy and protein). This study may inform considerations regarding instruments of choice in future population surveillance.

This trial was registered at Australian New Zealand Clinical Trials Registry as ACTRN12621000209897.

## Introduction

Surveillance of population dietary intakes provides vital data on nutritional adequacy to inform public health nutrition policy and programs [1]. The 24-h dietary recall (24HR) is currently the most commonly used method in population surveillance [[2], [3], [4], [5], [6]] because it is associated with lower bias than other dietary assessment methods feasible for large-scale studies [7]. Computer-based interviewer-administered 24HRs have been adopted in large-scale surveys [2,3] because they reduce participant and researcher burden. In multiple populations, technology-assisted 24HRs have been evaluated for accuracy prior to large-scale use [[8], [9], [10], [11], [12], [13], [14]], but few studies have conducted within-group comparisons among such methods relative to a measure of true intake.

A 24HR is designed to capture detailed information on foods and beverages consumed in the previous day or previous 24 h [15]. Recalling the details of previously consumed foods and beverages is a complex process [16,17]. Insights from cognitive psychology and formative research [18] have been used to inform strategies that enhance recall and reduce errors when recalling and reporting intake. For example, the interviewer-administered Automated Multiple-Pass Method (AMPM), used in the National Health and Nutrition Examination Survey in the United States, provides a structured interview format with specific probes using 5 sets or “passes”: a quick list, forgotten foods pass, time and occasion pass, detail pass, and final review [3,19]. Recently, a number of web-based interfaces based on AMPM have been developed to enable self-administration of 24HRs by participants, removing the need for highly-trained interviewers and coders [9,[20], [21], [22], [23]]. However, evaluation of the validity of self-administered 24HRs is critical to informing strategies to improve these methods [24].

24HRs are intended to measure total dietary intake of an individual in a single 24-h period. Thus, to assess whether 24HRs measure what they are intended to measure, i.e., their criterion validity, use of unbiased reference instruments is recommended as best practice [24]. A controlled feeding study design allows internal validation of a dietary assessment method to a reference method of true intake. These studies assess multiple components, such as food and beverage items served and consumed, with the quantities consumed measured from the direct weighing of items served minus any leftovers remaining. Indicators of total diet such as energy intake and macronutrient composition, as well as specific components of the diet such as intakes of micronutrients and food type and form, and portion size can be investigated [24]. Thus, controlled feeding studies provide high-quality, multidimensional information on total dietary intake during the study period. As intake during a controlled feeding study is short-term and highly controlled, it allows for evaluation of dietary assessment instruments independent of the many covariates and biases present in community-dwelling studies in which the dietary intakes of individuals are completely uncontrolled. If 24HR criterion validity (the measurement of total dietary intake) cannot be demonstrated for short-term intakes under highly controlled conditions, it follows that further refinement of the dietary assessment instrument is required prior to use in large-scale population studies. Thus, controlled feeding studies are a key methodology in assessment of 24HR validity.

The Automated Self-Administered Dietary Assessment Tool (ASA24), developed in the United States [20], and Intake24, developed in the United Kingdom [21,22], are 2 widely used self-administered 24HR methods that have been adapted for use in several countries, including Australia [25,26]. Local adaptation allows the tailoring of instruments to the target population, given the variation in dietary practices and culture across populations. Another way of using technology to potentially optimize the accuracy of 24HR is an image-assisted 24HR in which a participant collects real-time images of their foods/beverages before and after consumption and the images are available during the recall process. Using a combination of the images captured by participants and a 24HR is an under-investigated and relatively novel approach that may improve recall, reduce the cognitive burden on participants, and assist with food identification and portion size estimation [27]. In recent reviews, image-assisted approaches including 24HRs resulted in greater accuracy of self-reported dietary intake when compared with methods that did not use images supplied by participants [7,27,28]. In contrast to image-assisted dietary assessment, image-based dietary assessment uses images as the primary source of dietary data. Automated methods for image identification are rapidly evolving, but to date, most image-based food records are analyzed by a trained human analyst [27]. For example, for the mobile Food Record (mFR) app, a research dietitian viewed images taken by participants before and after eating. These images included a fiducial marker (a colorful checkered object of known size, color, and shape) that served as a reference for the dietitian when estimating portion size [29]. The accuracy of an image-assisted 24HR has yet to be compared with the mFR-Trained Analyst (mFR-TA) method, the web-based self-administered ASA24, or Intake24 in the same study population.

Thus, the aim of the current study was to compare the accuracy of energy and nutrient intake estimation of 4 technology-assisted dietary assessment methods [ASA24, Intake24, mFR-TA, and Image-Assisted Interviewer-Administered 24-h recall (IA-24HR)] relative to true intake across breakfast, lunch, and dinner in a sample of healthy adults aged 18 to 70 y in Australia.

## Methods

### Sample and recruitment

The details of the study protocol have been published previously [30] and are described briefly here. The sample was recruited in 2021 by advertising on the website and social media page of (Curtin University) and using snowball methodology (e.g., email newsletter and referrals from friends or colleagues). Participants were informed that the aim of the study was “to see how accurately people can estimate the amount of food and beverages consumed during a day and to work out which methods people prefer to use.” Quota sampling ensured equal numbers of men and women were recruited. To be included in the study, participants had to be able to attend in-person feeding sessions and have access to a computer and a smartphone (running iOS or Android OS) with a data plan. Respondents with serious illnesses or medical conditions; pregnancy; special dietary requirements; or dietary restrictions due to food allergies, intolerances, or dieting to lose weight were ineligible. Ethics approval from Curtin University Human Research Ethics Office was obtained (Approval number: HRE2019-0222) as was reciprocal ethics approval from the Department of Health Human Research Ethics Committee (Approval number: 201909.06). The study was registered with the Australian New Zealand Clinical Trials Registry (ACTRN12621000209897). All research design, practices, and reporting were aligned with the Australian Code for the Responsible Conduct of Research. Participants received a $20 (AUD) voucher for each of 3 in-person feeding sessions ($60 AUD maximum) as a token of appreciation for their involvement in the study.

### Study design

**FIGURE 1:**
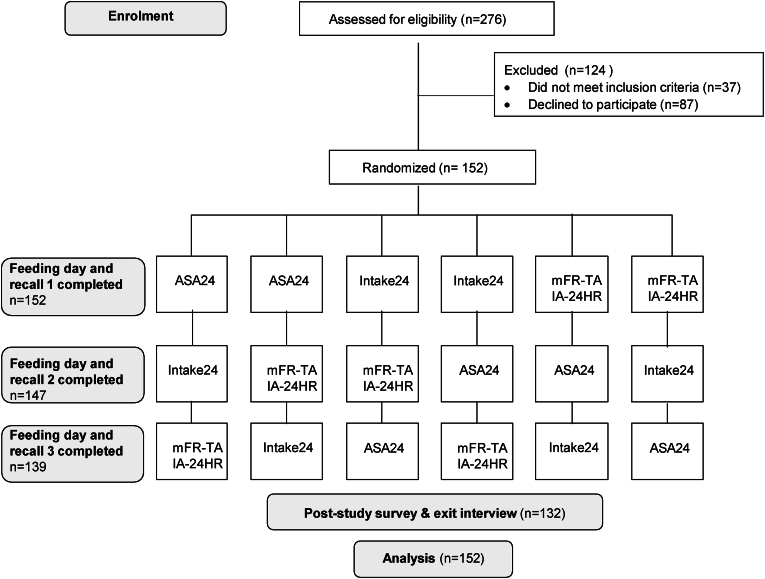
Study flow chart on enrollment, randomization, and study design. ASA24, Feeding day followed by completion of Automated Self-Administered Dietary Assessment Tool (ASA24)-Australia; Intake24, Feeding day followed by completion of Intake24-Australia; mFR-TA & IA-24HR, Feeding day including capture of images of meals using mobile Food Record app, followed by completion of Image-Assisted Interviewer-Administered 24-Hour Recall.

This controlled feeding study used a crossover design in which each of the 4 technology-assisted methods was used to assess 1 day of dietary intake for each individual: *1*) ASA24-Australia; *2*) Intake24-Australia; *3*) mFR-TA, and *4*) IA-24HR. The target sample size was 150 participants to allow for 20% drop-out while maintaining 90% power at a 5% significance level when the true difference between any 2 mean differences between estimated and true energy intake was 0. Participants undertook 3 feeding days (only 1 feeding day was required for the evaluation of mFR-TA and IA-24HR because the 2 methods used the same images). The sequence of the feeding days was randomized using a random number generator, stratified by gender, with at least a 1-wk washout period between each feeding day (Figure 1). On the first feeding day, height and weight were measured using standard protocols [31], and weight was measured on each subsequent feeding day.

### Procedures

Participants completed an online screening questionnaire and provided informed consent. Eligible participants were then asked to complete online demographic surveys and other psychosocial and cognitive measures [30]. Demographic characteristics, including age, gender identity, ethnicity, highest level of educational attainment, and annual household income, were recorded. Postcodes were collected to calculate the Index of Relative Socioeconomic Advantage and Disadvantage, a summary of social and economic conditions of individuals and households within an area [32].

### Feeding days

On each feeding day, participants selected from menus for each of breakfast, lunch, and dinner and consumed meals ad libitum. They were able to leave the laboratory between meals, and there were no restrictions on consumption of food and beverages between the meals, as the analysis protocol excluded all items reported as consumed outside the laboratory.

The study flow and menus were designed to represent a conventional eating environment [10,33] by providing foods that are commonly consumed in Australia [34] and comprised a mixture of cafe-style meals (e.g., curry and rice) and packaged products (e.g., yogurt, ice cream). There were 3 base menus that varied across the feeding days and that were adapted seasonally over the year and in response to the food supply on the day. Menu descriptions included details to enable participants to describe their intake using the recall methods, for example, the type of milk (e.g., full fat, skimmed). All menu items and descriptions for the study period are shown in Supplemental Table 1. Foods were served on a range of different plate and bowl sizes, and beverages were served in either ceramic cups or mugs, glass tumblers (large, medium, or small), or plastic cups. If a participant ordered salad dressing or condiments these were provided in separate containers so the weight consumed could be captured separately.

All food and beverage items were inconspicuously weighed in a separate laboratory space, using Kelba KHX-3 bench scales with 0.1g resolution, prior to being served to the participant on an individual tray. After eating, each tray was collected, and any leftover items were weighed. When necessary, leftover items were scraped from plates, separated, and placed on cling wrap for weighing. The amount eaten was determined by subtracting the weight of the plate waste from the weight of the served amount for each food. Weighing was conducted in duplicate, and a third measure was taken if the first 2 measures differed by >0.1 g [10]. An average of the 2 closest measures (rounded down to 1 decimal place) was recorded in a purposely-designed data sheet that was precoded with the 165 food codes from the Australian food nutrient database (AUSNUT 2011–13) [35] corresponding to the foods and beverages on the menu. Researchers took “before” and “after” images of all trays of food for future reference and image analysis.

### Dietary assessment methods

On each day following a feeding day, participants completed a 24HR remotely, using 1 of the 3 technology-assisted 24HRs (ASA24-Australia, Intake24-Australia, IA-24HR), consistent with the randomization schedule. The recalls were conducted remotely, to reduce face-to-face contact, in line with COVID-19 protocols in place at the time. During the IA-24HR feeding day, participants captured images of their meals using the mFR app. During the 24HRs, participants were asked to recall all foods and beverages consumed, including those consumed outside of the laboratory to adhere with standard 24HR protocols. In cases in which 24HRs were not completed, participants received reminders and were able to complete the 24HR ≤7 d later. The mFR-TA method was an image-based food record and used the images captured for IA-24HR. The steps involved in each dietary assessment method are shown in Table 1.

**TABLE 1:** Mapping of the USDA Automated Multiple-Pass Method interview structure to the 4 methods used in the study [the Automated Self-Administered Dietary Assessment Tool-Australia (ASA24), Intake24-Australia, the Image-Assisted Interviewer-Administered 24-hour recall (IA-24HR), and the mobile Food Record-Trained Analyst (mFR-TA)].

Automated Multiple-Pass Method steps	Aim/description	ASA24	Intake24	mFR-TA		Image-Assisted Interviewer-Administered 24HR	
				When image is taken	When image is not taken^1^	When image is taken	When image is not taken
Step 1: quick list	To obtain a quick report of foods and beverages consumed in the past 24 h without interrupting the participant.	Participant prompted to select an eating occasion and time, then conduct a free text search for matching food/beverage items, for each eating occasion in the previous 24 h.	Participant prompted to select an eating occasion and time and key in free text describing the items consumed in the previous 24 h. Participant then asked to select items returned by a search to match the free text.	—	—	Taken from the mini label and image provided by the participant; participant is asked to list any foods and beverages consumed that are not shown in images.	Participant asked to list all foods and beverages consumed.
Step 2: forgotten foods list and additions	To prompt the participant’s memory and collect other foods or beverages that are not reported in the quick list.	Participant prompted to review each gap between eating occasions and either report another meal or dismiss the gap.	Reported in steps 4 and 5.	—	—	Participant asked if they consumed items from a list of commonly forgotten foods.	Participant asked if they consumed items from a list of commonly forgotten foods.
Step 3: time and occasion	To record the time and occasion of food or beverages consumed.	Reported in step 1.	Reported in step 1.	—	—	Time of eating is recorded from the image metadata.	Participant asked to recall time and occasion of forgotten foods when item is reported.
Step 4: detail cycle	To collect specific descriptive information about each food item and beverage reported and record quantities and any additions made to the food.	Participant prompted to provide details of each food/beverage item (e.g., the form, preparation method, any additions, and the amount eaten). Standard food/beverage images assisted portion size estimation. Participant able to add food/beverages not included in the database, and add their own personal recipes, sandwiches, and salads by finding matching ingredients in the food list rather than selecting standard recipes.	Participant prompted to provide details of each food/beverage item (e.g., the form, preparation method, any additions/forgotten foods, and the amount eaten). Standard food/beverage images assisted portion size estimation. Participant able to add food/beverages not included in the database, and add their own personal recipes, sandwiches, and salads by finding matching ingredients in the food list rather than selecting standard recipes.	—	—	Participant asked to clarify only nonidentifiable food and beverage items; follow the Australian Health Survey Food Model Booklet to confirm amounts consumed; and check the after image for leftovers.	Participant asked food-specific probes to obtain details; follow the Australian Health Survey Food Model Booklet to confirm amounts consumed; and probed about leftovers.
Step 5: final probe	This is the last opportunity for the respondent to remember any new foods and beverages.	Participant prompted to review all foods and beverages selected, editing if necessary, to add any commonly forgotten foods and beverages they may have consumed, and to confirm they have recorded all of the food and beverages consumed on the previous day.	Participant prompted to review each gap between eating occasions, and either report another meal or dismiss the gap, to add any commonly forgotten foods and beverages they may have consumed, and to confirm they have recorded all of the food and beverages consumed on the previous day.	—	—	Read out the list of food and beverage items.	Read out the list of food and beverage items.
Data analysis	How responses are linked to food composition data.	Responses automatically linked to food composition data.	Responses automatically linked to food composition data.	Images captured by participant were analyzed, mini labels considered, and items coded into food composition software by trained analyst.	Images captured by researchers were analyzed and coded into food composition software by trained analyst.	Coded into food composition software manually by trained coder.	Coded into food composition software manually by trained coder.

1 In addition to participants capturing images, researchers also (covertly) took images of trays before and after eating, and these images were used by the trained analyst when the participant had not taken an image.

#### Automated Self-Administered Dietary Assessment Tool-Australia-2016 (ASA24)

Participants received a weblink, username, and password by email to access ASA24-Australia [25] and were asked to complete the 24HR by the end of the day. ASA24 includes passes adapted from the AMPM [20] (Table 1). The ASA24-Australia food and beverage database contains codes for >4800 foods/beverages from the Australian food nutrient database (AUSNUT 2011–13) [35].

#### Intake24-Australia

Participants received a personalized weblink via email to access Intake24-Australia [36] and were asked to complete the 24HR by the end of the day. A link to a 4-min instructional video embedded in the start page was provided for participants to watch before commencing Intake24-Australia, although it was not possible to track whether the video was viewed. Intake24-Australia includes passes adapted from the AMPM [21,37] (Table 1). The Intake24-Australia food and beverage database contained codes for >2800 foods/beverages from the Australian food nutrient database (AUSNUT 2011–13) [35].

#### Mobile Food Record

When allocated to IA-24HR, participants installed a mobile phone application called mFR on their smartphone on the feeding day prior to meals and were shown how to use it by researchers. The mFR application, an image-based dietary assessment system [[38], [39], [40], [41]], was then used to capture images of foods consumed during the specified feeding day. Participants were instructed to take “before” and “after” images of all foods and beverages consumed from the first meal served at the laboratory until midnight, including snacks not consumed as part of the study. They were asked to include a fiducial marker (a colorful checked object of known size, color, and shape that assists in food/beverage recognition and quantification) in each image [40]. The mFR automatically detected the presence of the fiducial marker and alerted participants if the fiducial marker was missing from an image. An angle-detection algorithm assisted participants to take the image at 45° to 60° from the horizontal plane. Once before and after images were captured, the images were automatically uploaded to the server. At the dinner session on the feeding day, prior to the meal, participants received training on a feature of the app known as the “Review” in which the images are returned to the participant to label the foods and beverages. The images were made available in the app for participants to review after midnight of the feeding day. To label a food or beverage, participants were asked to tap on the item, which caused a pin to appear. Tapping the pin took participants to the food list search function, where they conducted a free text search from a list of 372 food and beverage items. The food list was adapted for this study so that a mini label and short description were displayed to the participant. Once finished, the images with the confirmed pins were automatically sent to the server and disappeared from the application. Participants were asked to complete this labeling task prior to the scheduled IA-24HR interview the following day, during which they would refer to the images. The captured images were also used by a trained analyst to estimate and code dietary intake. When a participant had not labeled a food item but it was visible in the image, this was included in the analysis. If a participant did not capture the meal occasion, then the trained analyst accessed the researcher image for analysis. The training program for the analyst and the conduct of mFR-TA analysis are described in the data analyses section.

#### Image-assisted interviewer-administered 24-hour dietary recall (IA-24HR)

On the day after the feeding day, trained researchers conducted an interviewer-assisted 24-h recall with participants via a video call (protocol in Supplementary Material). The IA-24HR was interviewer-assisted to replicate the method used in the previous Australian Health Survey. Researcher training involved conducting 5 interviews subject to monitoring and feedback by the Principal Investigator. The researchers did not have access to the true intake data and were not present on feeding days. The interview followed an adapted multiple-pass approach (Table 1). Briefly, the quick list and time of eating were recorded by researchers prior to the interview using the labeled mFR images on the server. During the 24HR, the researcher used a screen-sharing function on the video call to enable both researcher and participant to simultaneously view each image. Participants were asked to provide food/beverage names and details when these were unclear from the images. Participants also provided portion sizes using household measures or the Food Model Booklet used in the Australian Health Survey [42]. The Food Model Booklet included images of common household food and beverage containers such as cups, glasses, and bowls, each with a known volume (in mL). It also contained images of mounds of various sizes with 3D rendering for describing amorphous foods (i.e., foods without a clearly defined form).

### Data analyses

Participant feeding days with ≥2 meals eaten at the food laboratory were included in analyses. Total energy and nutrient intakes (carbohydrate, total fat, protein, fiber, vitamin C, calcium, iron, potassium, folate) from each method and the controlled feeding sessions were calculated, excluding any items reported at eating occasions outside of the food laboratory, which were identified by manually examining eating times and eating occasions. Thus, total energy and nutrient intake refers to the sum of intake from meals consumed at the laboratory only. Macronutrient intakes as a percentage of total energy were calculated, to assess accuracy of macronutrient composition of the diet. Similarly, micronutrient intake per 1000 kJ was calculated. The macronutrients were intended to represent varying components of total diet. The micronutrients reported were intended to align with the selection of micronutrients reported in the Australian Health Survey. The same food composition database was used for all methods (AUSNUT 2011–13 food nutrient database [37]).

Protocols for data cleaning for ASA24-Australia [43] and Intake24-Australia [44] were followed. Checks for outliers in portion sizes and total energy and nutrient intakes were conducted to identify any obvious keying errors or food composition data anomalies; 6 were found and corrected before proceeding.

Analysis for mFR-TA followed a standard protocol for food identification and quantification using the fiducial marker. Human analyst training in food identification and quantification was conducted using data from a separate feeding study, and feedback was provided based on true weight data. Subsequently, for the current study, the trained analyst reviewed all before and after images captured by participants (or researchers when participant images were not available, e.g., images failed to upload). All consumed foods and beverages from breakfast, lunch, and dinner only were identified and quantified by the human trained analyst and entered into nutrition analysis software (FoodWorks 10, Xyris Software) linked to the AUSNUT 2011–13 food nutrient database [35] to estimate energy and nutrient intake for the 24HR period. The food and beverage labels added by participants during review were considered in the analyses. The trained analyst had access to the participant-labeled images through a website. This was conducted independently of participants. The trained analyst did not have access to the true food or beverage weights of items from this study.

Following IA-24HR, 2 coders followed instructions in a codebook and individually entered all IA-24HR recalls into nutrition analysis software (FoodWorks 10, Xyris Software) linked to the AUSNUT 2011–13 food nutrient database [35] to estimate energy and nutrient intake for the 24HR period. Food-specific density values from the AUSNUT 2011‒13 food measures database [45] were used to convert volumes derived from the Food Model Booklet into grams, but coder discretion was used to adjust derived amounts that they judged as conflicting with images. The double-coded data were compared with each another (total energy intraclass correlation coefficient (ICC) 0.987, *P* < 0.001), and all data entry errors were corrected by a single coder. The average (mean) of the 2 datasets was used in the analysis.

The average difference in item count (estimated – true item count) was determined for each method. For each method, the mean difference between true and estimated energy and nutrients was calculated along with the mean percentage difference between estimated and true intakes as (estimated – true) / true × 100. Mean differences were compared with 0 using 1-sample *t* tests. Point estimates and 95% confidence intervals (CIs) of the differences were used to consider whether evidence of differences between estimated and true intake was observed. Paired equality of variance tests using the SPSS MIXED procedure were used to assess differences in the variability of estimates between methods. Bland–Altman plots [46], using the true intake value on the x-axis [47], were generated to explore individual and group-level agreement between estimated and true intakes. Limits of agreement were plotted at ±1.96 SDs of the mean difference. Locally weighted scatterplot smoothing was conducted to examine trends in estimation error at varying levels of intake by identifying local or nonlinear trends in the data [48]. Within-person estimation error was assessed using Pearson correlation on the mean percentage difference between true and reported energy intake. The repeated measurements of mean difference and percentage mean difference were analyzed using the Linear Mixed Models procedure within SPSS Version 25, accounting for age, gender, and BMI (in kg/m^2^), with 24HR method and method order as fixed effects, to assess whether there were statistically significant differences by 24HR method. Age, gender, and BMI were included as covariates based on previously documented associations with the outcome variables [7,49,50]. Pairwise comparisons were conducted with Bonferroni adjustment for multiple comparisons to examine associations between each pair of methods. The STROBE-nut checklist (Supplemental Table 3) guided the reporting of this study [51].

## Results

### Participants

A total of 152 participants were randomized, and a total of 438 24HRs were completed. One hundred thirty-nine participants completed all 3 24HRs (*n* = 8 completed 2 24HRs and *n* = 5 completed 1 24HR and were included in the analysis owing to the crossover study design). The median (25th, 75th percentiles) washout period was 7 d between feeding days 1 and 2 (7, 7 d) and 2 and 3 (7, 8 d). Nine participants had a 5-d washout period between feeding days 2 and 3 to accommodate the study end date. Self-administered recalls were typically completed in under 30 min with median (25th, 75th percentiles) as follows: for ASA24, 26 min (18, 37 min) and Intake24, 19 min (13, 35 min). The interview-administered IA-24HR method was 18 min (15, 21 min). Slightly more women than men took part in the study (Table 2). More than half (56%) of participants identified as being of Asian ethnicity, and almost three-quarters (71%) were educated to university degree level or higher. The mean age of participants was 32 y (SD 11 y; range 18–76 y), with few participants (*n* = 4) aged ≥60 y. Almost half of the participants (42%) lived in areas of relative socioeconomic advantage. The average difference (± SD) between estimated and true item count was 2.2 ± 2.5 (IA-24HR), 1.0 ± 5.0 (ASA24), −1.0 ± 4.4 (Intake24), and −0.5 ± 1.4 (mFR-TA).

**TABLE 2:** Demographic characteristics of participants (*n* = 152) of controlled feeding study.

Characteristic	Summary statistics
Age, y, mean (SD)	32 (11)
BMI, kg/m^2^, mean (SD)	26 (5)
Gender identity, *n* (%)	
Woman	84 (55.3%)
Man	63 (41.4%)
Prefer not to say	5 (3.3%)
Ethnicity, *n* (%)	
White	59 (38.8%)
Aboriginal or Torres Strait Islander	1 (0.7%)
Asian	85 (55.9%)
Black or African American	1 (0.7%)
Other	6 (3.9%)
Annual household income ($ AUD), *n* (%)	
<$60,000	41 (27.0%)
$60,000–$149,999	63 (41.4%)
$150,000 or above	30 (19.7%)
Don't know, or prefer not to answer	18 (11.8%)
Highest level of education attained, *n* (%)	
School or diploma	44 (28.9%)
University bachelor’s degree or higher	108 (71.1%)
Index of relative socioeconomic advantage and disadvantage, *n* (%)	
Quintile 1 (most disadvantaged)	1 (0.7%)
Quintile 2	36 (23.8%)
Quintile 3	26 (17.2%)
Quintile 4	25 (16.6%)
Quintile 5 (least disadvantaged)	63 (41.7%)

### Energy intake estimation error

In this study, true intake was based on the sum of items consumed at breakfast, lunch, and dinner on the feeding day. There was no evidence of a difference between true and estimated energy intakes using ASA24-Australia (mean difference: 319 kJ; 95% CI: −98, 736 kJ), Intake24-Australia (−110 kJ; 95% CI: −494, 274 kJ), and mFR-TA (−56 kJ; 95% CI: −252, 141 kJ) (Table 3). Variance of estimated energy intake was statistically significantly higher than the variance of true intake for ASA24 and IA-24HR and lower than the variance of true intake for mFR-TA (all *P* < 0.01). No differences in variance were detected between estimated and true intakes using Intake24 (*P* = 0.1). Using IA-24HR, estimated energy intake was higher than true intake (1180 kJ; 95% CI: 907, 1453 kJ). Considering the mean difference as a percentage of true intake, estimates were within 6% of true intake using ASA24-Australia (5.4%; 95% CI: 0.6, 10.2%), Intake24-Australia (1.7%; 95% CI: −2.9, 6.3%), and mFR-TA (1.3%; 95% CI: −1.1, 3.8%). In contrast, IA-24HR estimates were higher than true intake by 15.0% (95% CI: 11.6, 18.3%) (Table 3).

**TABLE 3:** Descriptive statistics of estimated and true energy and nutrient intake, by dietary assessment method.

	ASA24 *n* = 143				Intake24 *n* = 150				mFR-TA *n* = 148				IA-24HR *n* = 145			
	Estimated intake	True intake	Difference	P	Estimated intake	True intake	Difference	P	Estimated intake	True intake	Difference	P	Estimated intake	True intake	Difference	P
	Mean (SD)	Mean (SD)	Mean (95% CI)		Mean (SD)	Mean (SD)	Mean (95% CI)		Mean (SD)	Mean (SD)	Mean (95% CI)		Mean (SD)	Mean (SD)	Mean (95% CI)	
**Energy**																
kJ	8927 (3804)	8608 (3128)	319 (−98, 736)	0.13	8550 (3399)	8659 (3180)	−110 (−494, 274)	0.57	8628 (2955)	8684 (3220)	−56 (−252, 141)	0.58	9802 (3731)	8622 (3183)	1180 (907, 1453)	<0.001
As % of true intake	105 (29)	100 (0)	5.4 (0.6, 10.2)	0.03	102 (29)	100 (0)	1.7 (−2.9, 6.3)	0.47	101 (15)	100 (0)	1.3 (−1.1, 3.8)	0.28	115 (20)	100 (0)	15.0 (11.6, 18.3)	<0.001
**Carbohydrate**																
g	227.1 (102.1)	227.0 (87.0)	0.1 (−11.0, 11.2)	0.99	241.6 (98.5)	233.2 (88.1)	8.4 (−2.1, 18.9)	0.12	229.6 (81.6)	234.9 (91.9)	−5.3 (−11.1, 0.5)	0.08	260.5 (100.1)	233.1 (91.4)	27.4 (19.9, 34.9)	<0.001
As % total energy	42.8 (8.9)	43.9 (5.6)	−1.16 (−2.46, 0.15)	0.08	47.7 (8.8)	45.1 (6.2)	2.55 (1.42, 3.67)	<0.001	44.6 (7.2)	45.1 (7.0)	−0.5 (−1.2, 0.2)	0.16	44.6 (7.6)	45.1 (7)	−0.5 (−1.24, 0.24)	0.18
**Protein**																
g	109.0 (55.4)	107.3 (47.4)	1.7 (−4.4, 7.8)	0.59	94.6 (48.5)	106.2 (50.1)	−11.6 (−17.9, −5.3)	<0.001	105.3 (45.4)	106.4 (48)	−1.2 (−4.6, 2.3)	0.51	114.4 (54.4)	105.3 (47.0)	9.0 (4.4, 13.6)	<0.001
As % total energy	20.7 (6.3)	20.7 (4.6)	−0.04 (−0.83, 0.74)	0.91	18.4 (4.8)	20.2 (4.6)	−1.8 (−2.42, −1.17)	<0.001	20.2 (4.8)	20.3 (4.6)	−0.1 (−0.6, 0.3)	0.54	19.3 (5.1)	20.3 (4.6)	−0.96 (−1.44, −0.49)	<0.001
**Total fat**																
g	88.1 (42.9)	74.2 (28.9)	13.9 (8.8, 19.1)	<0.001	72.1 (34.3)	73.2 (28.8)	−1.1 (−5.4, 3.2)	0.62	74.2 (29.4)	73.1 (30.2)	1.1 (−1.5, 3.7)	0.42	86.6 (37.6)	72.8 (30.0)	14.0 (10.9, 17.1)	<0.001
As % total energy	37.0 (9.0)	32.6 (5.4)	4.39 (3.12, 5.67)	<0.001	31.3 (7.0)	32.0 (5.2)	−0.61 (−1.61, 0.39)	0.23	32.4 (6.1)	31.8 (6.1)	0.5 (−0.1, 1.2)	0.09	33.3 (6.1)	31.9 (6.1)	1.35 (0.73, 1.97)	<0.001
**Fiber**																
g	25.3 (11.6)	24.9 (8.9)	0.5 (−1.0, 1.9)	0.53	23.8 (10)	25.7 (9.5)	−1.8 (−3, −0.6)	0.004	25.5 (9.5)	25.2 (9.5)	0.3 (−0.4, 1.0)	0.35	29.0 (11.5)	24.9 (9.3)	4.1 (3.0, 5.2)	<0.001
g/1000 kJ	3.0 (1.0)	3.0 (0.9)	0.0 (−0.2, 0.1)	0.46	2.8 (0.8)	3.1 (0.9)	−0.2 (−0.3, −0.1)	<0.001	3.0 (0.8)	3.0 (0.9)	0.0 (0.0, 0.1)	0.60	3.0 (0.9)	3.0 (0.9)	0.0 (0.0, 0.1)	0.27
**Vitamin C**																
mg	197.5 (140.8)	178.4 (125.4)	19.1 (5.8, 32.3)	0.005	174.9 (142.6)	188.6 (126.8)	−13.7 (−28.6, 1.2)	0.071	211.9 (164)	182.7 (133)	29.2 (19.5, 38.9)	<0.001	226.3 (159.4)	181.6 (133.8)	44.6 (35.5, 53.8)	<.001
mg/1000 kJ	23.1 (15.8)	21.7 (13.9)	1.4 (−0.4, 3.2)	0.13	21.1 (19.1)	22.9 (14.9)	−1.7 (−3.8, 0.4)	0.106	25.1 (18.1)	22.3 (16.2)	2.9 (1.8, 4)	<0.001	23.9 (16.4)	22.2 (16.2)	1.7 (0.7, 2.6)	<0.001
**Calcium**																
mg	842.0 (457.3)	779.2 (333)	62.7 (8.5, 117)	0.02	779.3 (424)	789.2 (351.4)	−9.9 (−53.2, 33.4)	0.651	755.6 (345.7)	771.6 (345.9)	−16 (−38.7, 6.7)	0.17	843.8 (400.2)	762.2 (341.7)	81.6 (51.5, 111.7)	<0.001
mg/1000 kJ	97.0 (41.5)	94.8 (41.9)	2.1 (−3.0, 7.3)	0.42	91.4 (36.3)	93.8 (36)	−2.4 (−6.1, 1.3)	0.206	89 (33.9)	92.1 (34.5)	−3.1 (−6.1, 0)	0.05	88.4 (33.7)	91.7 (34.6)	−3.3 (−6.4, −0.1)	0.04
**Potassium**																
mg	3543.8 (1551.5)	3367.6 (1179.8)	176.2 (20.5, 331.9)	0.03	3082.6 (1239.1)	3388.3 (1228)	−305.8 (−459.1, −152.5)	<0.001	3518 (1272.4)	3433.7 (1212.2)	84.3 (−1.6, 170.3)	0.05	4046.6 (1642.3)	3406.1 (1193.1)	640.5 (498.6, 782.3)	<0.001
mg/1000 kJ	406.6 (109.4)	401.8 (85.9)	4.8 (−8.2, 17.8)	0.47	365.8 (84.5)	400.2 (86.4)	−34.4 (−46, −22.8)	<0.001	412.0 (83.0)	406.1 (84.7)	6.0 (−0.9, 12.8)	0.09	417 (90.5)	405.9 (85.3)	11.1 (2.7, 19.6)	0.01
**Iron**																
mg	11.3 (5.4)	10.6 (4.1)	0.7 (0.1, 1.3)	0.03	10.3 (5.2)	11 (4.5)	−0.7 (−1.3, −0.1)	0.025	11.2 (4.7)	11.2 (5.4)	0.0 (−0.5, 0.4)	0.95	12.0 (5.1)	11.1 (5.4)	0.9 (0.4, 1.5)	0.001
mg/1000 kJ	1.3 (0.3)	1.3 (0.3)	0.0 (0.0, 0.1)	0.22	1.2 (0.3)	1.3 (0.3)	−0.1 (−0.1, 0)	<0.001	1.3 (0.3)	1.3 (0.4)	0.0 (−0.5, 0.4)	0.89	1.2 (0.3)	1.3 (0.4)	−0.1 (−0.1, 0.0)	<0.001
**Folate**																
μg	588.4 (244.3)	601.1 (231.9)	−12.7 (−43.3, 18.0)	0.42	571.2 (269.0)	632.4 (269.1)	−61.3 (−93.6, −29.0)	<0.001	580.8 (230.1)	604.7 (259.6)	−23.9 (−43.8, −4.1)	0.02	625.4 (234.9)	598.8 (258.7)	26.6 (2.9, 50.3)	0.028
μg/1000 kJ	69.6 (22.1)	71.8 (18.8)	−2.2 (−5.6, 1.2)	0.20	68.0 (21.1)	73.7 (18.8)	−5.8 (−8.7, −2.8)	<0.001	68.8 (19.4)	71.4 (20.7)	−2.7 (−4.5, −0.8)	<0.001	66.4 (21.2)	71.2 (20.4)	−4.7 (−6.9, −2.6)	<0.001

True intake, Intake calculated from food weights in controlled feeding laboratory.

Mean difference = (reported intake – true intake) / true intake × 100.

*P* values derived from 1 sample *t* tests of mean difference.

Abbreviations: 95% CI, 95% confidence interval of the mean differences; ASA24, Automated Self-Administered Dietary Assessment Tool-Australia; CI, confidence interval; IA-24HR, Image-Assisted Interviewer-Administered 24-hour recall; mFR-TA, mobile Food Record-Trained Analyst; SD, standard deviation.

**FIGURE 2:**
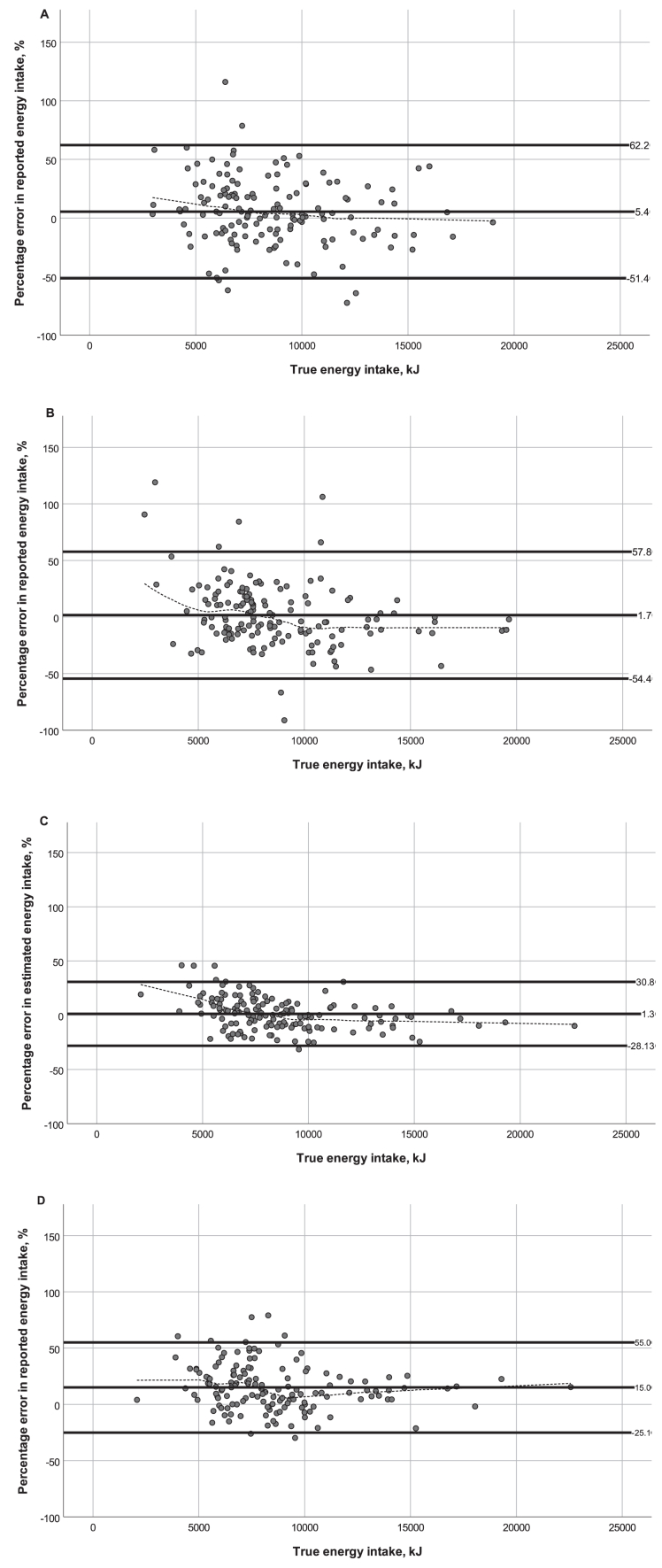
Bland–Altman scatterplots of percentage error in estimated energy intake against true energy intake, showing mean difference and upper and lower limits of agreement (± 1.96 standard deviation) (solid lines), and locally weighted scatterplot smoothing lines (dotted lines). (A) ASA24-Australia; (B) Intake24-Australia; (C) mFR-TA; (D) IA-24HR. ASA24, Feeding day followed by completion of Automated Self-Administered Dietary Assessment Tool (ASA24)-Australia; Intake24, Feeding day followed by completion of Intake24-Australia; mFR-TA & IA-24HR, Feeding day including capture of images of meals using mobile Food Record app, followed by completion of Image-Assisted Interviewer-Administered 24-Hour Recall.

Bland–Altman plots (Figure 2) illustrated broad limits of agreement, which were close to ±50% for ASA24-Australia and Intake24-Australia, but within 30% for mFR-TA. Visual interpretation of locally weighted scatterplot smoothing lines (Figure 2) suggested that based on Intake24-Australia, energy intake of individuals with lower true intakes was overestimated and energy intake of individuals with higher true intakes was underestimated. A similar pattern was observed for ASA24-Australia and mFR-TA, but to a lesser extent (Figure 2). In contrast, total energy intake estimated using IA-24HR was higher than true intake across the range of true energy intakes. An association was detected between percentage error in energy intakes estimated using ASA24-Australia and Intake24-Australia (*r* = 0.17, *P* = 0.04) and between percentage error in energy intakes estimated using mFR-TA and IA-24HR (*r* = 0.53, *P* < 0.001). The proportion of participants with estimated energy intake within 800 kJ of true intake was highest using mFR-TA (45%) and similar among other methods (29%–34%) (Supplemental Figure 1).

Recall method, but not method order, was statistically significantly associated with the mean difference between estimated and true energy intake (kJ and percentage, both *P* < 0.001), controlling for gender, BMI, and age. Pairwise comparisons indicated this result was driven by IA-24HR as there was a significant difference in mean differences between IA-24HR and all other methods (*P* < 0.01, for mean difference, kJ and percentage), whereas no differences were detected among any of the other methods.

### Nutrient intake estimation error

Error in the assessment of all nutrients varied by recall method. As with total energy, recall method, but not method order, was associated with the difference between true and estimated intakes for all nutrients (all *P* < 0.001), after controlling for gender, age, and BMI.

#### Macronutrients

True macronutrient intake was based on the sum of items consumed at breakfast, lunch, and dinner on the feeding day. Using mFR-TA, there was no evidence of a difference between estimated and true intake of any macronutrient, either in grams or as a percentage of total energy (Table 3), but the variance of estimated carbohydrate (*P* < 0.001) and protein (*P* = 0.06) intake was statistically significantly lower than the variance of true intake. With the other methods, differential accuracy in estimation of macronutrients was present. There was no evidence of a difference between estimated and true carbohydrate and protein intakes in grams or as a percentage of total energy using ASA24-Australia, whereas estimated fat intake (g) was higher than true intake (13.9 g; 95% CI: 8.8, 19.1 g) (Table 3). The variances of estimated carbohydrate, protein, and total fat intake using ASA24 were significantly higher than the variances of true intake (all *P* < 0.001). In contrast, using Intake24-Australia, there was no evidence of a difference between estimated and true fat intake or between the variances of estimated and true protein intake (*P* = 0.3), although estimated protein intake was lower than true intake (−11.6 g; 95% CI: −17.9, −5.3) (Table 3). Using IA-24HR, estimated carbohydrate, protein, and fat intakes were higher than true intakes, but there was no evidence of a difference between estimated and true carbohydrate intake as percentage of total energy. The variances of estimated carbohydrate, protein, and total fat intake using IA-24HR were significantly higher than the variances of true intake (all *P* < 0.001).

The percentage error in estimated carbohydrate and protein intake was <10% using ASA24-Australia, Intake24-Australia, and mFR-TA, but broad limits of agreement (more than ±50%) were observed for both ASA24-Australia and Intake24-Australia (Supplemental Figures 2 and 3). In contrast, the mean error of IA-24HR was 14%, but the limits of agreement were narrower (−25.6%, 52.5%). Using mFR-TA, the limits of agreement were narrowest compared with other methods, for all macronutrients (Supplemental Figures 2–4).

Investigations across levels of intake using locally weighted scatterplots indicated that, in contrast to total energy intake, carbohydrate intake seemed to be estimated to a similar level of accuracy at all levels of intake (i.e., both high and low true intakes) by ASA24-Australia and Intake 24-Australia. However, the scatterplots indicated higher estimated protein and fat intakes among individuals with lower true intakes and lower estimated protein and fat intake among individuals with higher true intakes using Intake24-Australia (Supplemental Figures 3 and 4).

There was no evidence of a difference between estimated and true fiber intake using ASA24-Australia (0.5 g; 95% CI: −1.0, 1.9 g) and mFR-TA (0.0 g; 95% CI: 0.0, 0.1 g). Estimated fiber intake was slightly lower than true intake using Intake24-Australia (−1.8 g; 95% CI: −3.0, −0.6 g) and slightly higher than true intake using IA-24HR (4.1 g; 95% CI: 3.0, 5.2 g). There was little evidence of a difference between estimated and true energy-adjusted fiber intakes for all methods (Table 3).

#### Micronutrients

Estimated absolute intakes of vitamin C, calcium, potassium, and iron were slightly higher than true intakes using ASA24-Australia, but there was no evidence of a difference between energy-adjusted micronutrient estimates and true intakes (Table 3). In contrast, using Intake24-Australia, there was no evidence of a difference between estimated intakes of vitamin C and calcium, but estimated absolute and energy-adjusted intakes of potassium, iron, and folate were lower than true intakes. Using mFR-TA, estimated absolute and energy-adjusted intakes of vitamin C were higher than true intakes, whereas estimated intakes of folate were lower than true intakes. There was no evidence of a difference between estimated intakes of calcium, potassium, and iron using mFR-TA. Estimated absolute intakes of all micronutrients were higher than true intakes using IA-24HR.

## Discussion

In the current study, we aimed to compare the accuracy of 4 technology-assisted dietary assessment methods in estimating energy and nutrient intakes relative to true intake in a controlled feeding study. For ASA24-Australia, Intake24-Australia, and mFR-TA, there was no evidence of differences between estimates of group-level absolute energy intake and true intake, but between-method differences existed in the estimation of nutrient intakes. Overall, using mFR-TA provided estimates of total energy and nutrient intake that most consistently aligned with true average intakes, but Intake24 estimates were best aligned with the true intake distribution. We found that using IA-24HR, in which participants completed an interviewer-administered 24HR the following day, resulted in estimates of total energy intake and absolute intake of all nutrients that were substantially higher than true intakes. Energy-adjusted micronutrient intakes were consistently estimated accurately by ASA24-Australia and mFR-TA but not by the other methods. Across methods, carbohydrate appeared to be the most accurately estimated macronutrient, whereas total fat appeared to be the least accurately estimated, but there is a general lack of guidance on what constitutes a reasonable level of accuracy, so some of these differences may not be meaningful.

The nutrient density method, which considers macronutrients as a percentage of energy intake rather than absolute amounts, did not substantially improve the methods’ performance in the estimation of macronutrient intake, except for IA-24HR in which substantial overestimation of energy intake occurred. In the current study, both ASA24-Australia and Intake24-Australia assessed absolute total energy intake more accurately than macronutrient intakes. Similarly, in a controlled feeding study evaluating the accuracy of ASA24 among 302 United States women with low incomes, no evidence of a difference was observed between estimated and true average energy intake, whereas protein intake was underestimated [52]. To date, no validation studies have been conducted to evaluate the performance of Intake24-Australia in the estimation of macronutrient intake using unbiased references measures, such as controlled feeding studies or biomarkers. However, a study using doubly labeled water to estimate total energy expenditure among 98 United Kingdom adults found that Intake24 estimated energy intake to be 25% lower on average than true intake [53], whereas in the current study, there was no meaningful difference between true energy intake and energy intake estimated by Intake24. This contrast in findings according to study design (biomarker versus controlled feeding) may be a result of the different measures of interest (usual intake versus intake on a single day). Future investigations into how well the methods in the current study can assess usual intake are warranted.

Differences in the assessment of nutrient intake were observed between the 2 self-administered 24HRs used in this study. Despite the similarity of the ASA24-Australia and Intake24-Australia platforms, differences include the type and number of probing questions and the variety and number of portion size images available. These platform differences may have contributed to the differential findings of this study. A recent qualitative “think-aloud” study among students in Australia evaluated usability of the ASA24-Australia and Intake24-Australia dietary assessment tools to compare problems encountered when completing the 2 24HRs. Problems were categorized as “remembering,” “program,” and “emotion” [54]. In this direct comparison of the 2 platforms, participants reported almost twice as many perceived problems during the completion of ASA24-Australia than with Intake24 [54]. Differences in user experience may have contributed to the differences observed between ASA24-Australia and Intake24-Australia in the current study, as well as in the think-aloud study [54]. Examining and comparing system usability and acceptability across platforms and how this relates to accuracy is recommended to inform the further development and refinement of self-administered 24HRs.

In the current study, using mFR-TA resulted in estimates of most nutrients that were not meaningfully different from true intakes. In a meta-analysis investigating the validity of image-based dietary assessment, 13 studies were identified but none used a controlled feeding study design [55], so direct comparison cannot be made. Several previous studies using doubly labeled water to evaluate a trained analyst approach to image-based dietary assessment show the method’s ability to assess habitual dietary intake [56,57]. For example, in a study of 45 community-dwelling adults, energy intakes estimated by trained analysts using mFR images over a 7.5-d period were 80% of total energy expenditure for this period based on doubly labeled water, indicating that the method was comparable to other image-based and traditional dietary assessment methods [57]. The contrast in findings between mFR-TA and IA-24HR, which involved the same participants, the same true intake, and the same images, provides a clear indication of the added value of a trained analyst. For the mFR-TA, a consideration is the additional cost for the trained analyst to conduct the dietary analyses from the images. These results also suggest there is no added benefit of the interview, potentially reducing the cost and participant and researcher burden. Using IA-24HR, food and beverages were identified by interviewers and confirmed by participants, but the key difference between IA-24HR and mFR-TA is that the trained analyst estimated portion sizes. Portion sizes in IA-24HR were estimated using household measures for single items or standard amounts (e.g., a banana, a slice of bread) and a Food Model Booklet used in the Australian Health Survey [42]. This booklet included images of cups, glasses, bowls, and 3D-rendered mounds for foods and beverages that were hard to describe and often amorphous (e.g., a mound of rice). Participants were served on disposable plates that varied in size from those illustrated in the Food Model Booklet. For example, the disposable dinner plate was 23 cm (9 inches) whereas the dinner plate in the booklet to estimate their portion sizes was 27 cm in diameter. This may have contributed to an overestimation of portion sizes because plate size is directly associated with self-served portion size [58]. Automatic quantification methods of food images collected by participants, including volume estimation, continue to be developed using computer vision and machine learning techniques [39,41,59]. Such approaches have the potential to refine and improve portion size assessment in methods such as IA-24HR [[60], [61], [62]]. As development of such methods continues, the use of trained analysts will remain vital in image-based dietary assessment and is recommended.

The purpose of large-scale surveillance of population dietary intakes is to track progress toward population dietary guidelines and identify which population subgroups have inadequate intakes to inform both policy and practice related to optimizing dietary patterns and nutritional health. Such surveillance requires methods that assess the usual or habitual intake at the group or population level with reasonable accuracy, as this is the measure of relevance to health [63]. Despite a lack of consensus on what constitutes a reasonable level of accuracy [64], our findings that Intake24-Australia, ASA24-Australia, and mFR-TA estimated energy intakes to within 6% of true intakes suggest that these methods are able to reasonably estimate population average values of total dietary intake under controlled conditions. It should be noted that the sample distribution of intake estimated by each method did not reflect the distribution of true intakes, except when using Intake24. Accurate estimation of intake distributions is important when information is required on the proportion of a population above or below a certain level of intake. To obtain a reliable distribution of usual population dietary intakes, day-to-day variation must be accounted for, by making repeat measurements, for example [63]. Although a single day of data cannot provide information on an individual’s usual or habitual dietary intake due to within-person variation in dietary intake, the current study has taken an initial step in evaluating, under controlled conditions, the criterion validity of a single day of methods used in population surveillance. Further research is needed to confirm if these findings can be replicated in more diverse community-dwelling populations.

Our self-selecting sample is unlikely representative of the general population in Australia. Although our original protocol involved recruitment from the electoral role, due to campus COVID-19 restrictions at the time of the study, only university staff and students with access to the campus were able to participate. As such, the sample had higher educational attainment, was younger on average, and had lower social disadvantage, and the proportion of Asian individuals was higher than that in the general population in Australia [65]. The 24HR methods may perform differently in a different group with different characteristics, and this requires further investigation. Although the current study findings may not be generalizable to the wider population, they support the continued investigation and evaluation of these methods in the Australian population to enable ongoing high-quality surveillance.

The controlled feeding design used in the current study, a relatively uncommon design among dietary validation studies, allowed for assessment and understanding of the criterion validity of various dietary assessment methods. To our knowledge, this is the first study using a crossover design to evaluate differences in these 24HR methods, including an image-based dietary assessment method. The crossover design provides confidence that differences observed between methods are not attributable to differences in participant characteristics and behaviors. In studies using repeated measurements, it is possible that participants may report intake with increasing or decreasing accuracy at each subsequent recall. For example, after the first feeding session and recall, participants would be able to guess that they would be asked about their food intake after the second and third recall and thus may have paid more attention to the items consumed. However, the order in which methods were administered was randomized and not associated with accuracy.

In the current study, the menu comprised a variety of foods and was rotated each week to ensure variety between feeding days. Although the study included packaged products, the proportion of packaged products consumed in this study may have been lower than in community-dwelling settings. This may have resulted in an underestimation of accuracy in the methods assessed, given that more specific food composition data are available for branded products compared to generic items. Furthermore, despite our effort to replicate a real-world café setting, it was not possible to fully represent the vast variety of foods available in community settings. In contrast, the absence of snacks from the controlled feeding environment may have resulted in more accurate estimates of total intake, given that omission of snacks from 24HR has been observed [9,66]. The novelty of the controlled feeding environment may cause participants to be more aware of the foods they consumed and therefore report more accurately. Therefore, the findings observed under these controlled conditions may not be generalizable to the general population. The presence of researchers to remind participants to capture images using the mFR app meant that the issue of forgetting to take images was likely to be lower than it would have been under less controlled conditions. Previous work with the mobile food record has shown remembering to take images before snacks was more difficult than before meals [67]. The interviewer-assistance given with the IA-24HR method may have introduced additional social desirability bias when recalling intake compared to the other methods. With mFR-TA, the trained analyst was an experienced research dietitian, but this may not reflect personnel resources available in future large-scale studies. More broadly, despite its accuracy, the mFR-TA approach may be less feasible due to resource constraints in large-scale studies than the self-administered methods. With fully automated methods for food identification and portion size estimation not yet available, robust manual methods are still needed.

Differences observed in the item count (estimated – true item count) between methods could indicate omissions of consumed items, intrusions of unconsumed items, or differences in data entry protocols for IA-24HR for multicomponent dishes (e.g., “mixed vegetables” versus “broccoli” + “peas” + “corn”). Therefore, any differences in item count may possibly be due to the nature of the recall systems and whether they facilitate reporting multi-ingredient items together or separately.

In Australia to date, there has been no validation work with the methods used in national surveys. Unfortunately, no validation study design is able to address all research questions, but the current study is an important first step, despite its limitations, in providing insights into method accuracy under controlled conditions. Evaluating these methods under less controlled conditions in more diverse community-dwelling populations to establish the external validity is a future direction for this research, although validation of food and beverage types and amounts remains a challenge. Other approaches to assessing true intake can add to the overall evaluation of dietary assessment methods used in Australian national surveys, such as the duplicate plate method, or collection of biomarkers which, despite their limitations, may offer insights into how well dietary assessment methods assess intake in community-dwelling environments.

In conclusion, this controlled feeding study evaluated 4 technology-assisted dietary assessment methods for accuracy in estimating energy and nutrient intakes based on 3 meals consumed in a controlled environment. The results suggest that Intake24-Australia, ASA24-Australia, and mFR-TA have reasonable validity for estimation of population dietary intakes but that further investigation is needed into the drivers of estimation error for IA-24HR. With advances in automated methods, image-assisted methods such as those used in this study may lead to improvements in accuracy while reducing participant burden and cost. The data in the current study enables the understanding of the rapidly evolving landscape of technology-assisted dietary assessment, to inform decisions for surveillance.

## Acknowledgments

We thank all the study participants for contributing their time to this research, the research assistants for supporting data collection (Clare Keating, Lani Le, Sophie Bell, Sophy Tremain-Hill, Lisa Goldsworthy, Li Ling Loke, and Ee Zin Woon), and the mFR engineers (Jiangpeng He, Vinod Gautham, and Zeman Shao). We acknowledge Western Australia Data Linkage Branch, Department of Health, Western Australia for providing Western Australian Electoral Roll sample data. The data were requested and provided but not utilized in the recruitment of participants for this study.

## Author contributions

The authors‘ responsibilities were as follows – DAK, CJB, CEC, BAM, MER, SSD, RN, EJD, FZ, SIK, PA, CMP: conceived the research question and overall design of the study; DAK, BAM, RN, CJB, CEC, MER, TAM, JDH, CW, CJB, EJD, FZ: planned and compiled measurement tools, and protocols; DAK, SSD, MER, SAM, CW: planned the data management and analysis; CW, JDH, AH, SAM, SG, DAK: collected the data; CW, SG: conducted data entry; CW: cleaned and analyzed the data; CW: drafted and edited the manuscript; and all authors: revised the manuscript for intellectual content and read and approved the final manuscript.

## Conflict of interest

DAK, CJB, and EJD hold 2 patents for the mobile Food Record as follows: C. Boushey, E.J. Delp, D.S. Ebert, K.D. Lutes, D. Kerr, “Dietary Assessment System and Method,” U.S. Patent 8 605 952 B2, December 10, 2013; C. Boushey, E.J. Delp, D.S. Ebert, K.D. Lutes, D. Kerr, “Dietary Assessment System and Method,” U.S. Patent 8 363 913 B2, January 29, 2013. All other authors report no conflicts of interest.

## Funding

This study was funded by an Australian Research Council Discovery Project 190101723 entitled “Accuracy and cost-effectiveness of technology-assisted dietary assessment.” ASA24, the Automated Self-Administered (ASA24) Dietary Assessment Tool, version 2016, was developed by the National Cancer Institute, Bethesda, MD. ASA24 is a registered trademark of HHS. In collaboration with the National Cancer Institute (NCI), a consortium of Australian researchers adapted ASA24 to the Australian context, led by The Institute for Nutrition and Physical Activity (IPAN) at Deakin University. Intake24 is an open-source web-based dietary assessment research tool based on the 24-h recall method, primarily designed for self-completion. Intake24 was created by Newcastle University (UK), funded by Food Standards, Scotland. The system is further developed and maintained through a collaboration between Cambridge University, Monash University, and Newcastle University (UK). The mobile Food Record app study was funded by NIH-NCI (1U01CA130784-01, 1U24CA268228-01) and NIH-NIDDK (1R01DK073711-01A1, 2R56DK073711-04). The term mobile Food Record (mFR) is a registered trademark. CW and JDH are supported by an Australian Research Training Programme scholarship. CEC is supported by a National Health and Medical Research Council of Australia Leadership (L3) Research Fellowship (APP2009340). The sponsors had no role in the design of the study; in the collection, analyses, or interpretation of data; in the writing of the manuscript, and in the decision to publish the results.

## Data availability

Data described in the manuscript, code book, and analytic code will not be made available because secondary analyses are yet to be reported.
