# Supplementary material for: Accuracy of energy and nutrient intake estimation versus observed intake using 4 technology-assisted dietary assessment methods: a randomized crossover feeding study
Source: Am J Clin Nutr. 2024 May 6;120(1):196–210. doi: 10.1016/j.ajcnut.2024.04.030 (PMC11347807; doi:10.1016/j.ajcnut.2024.04.030)
Supplement: Multimedia component 1 [file mmc1.pdf]

Whitton et al. Accuracy of energy and nutrient intake estimation versus observed intake using 4 technology assisted dietary assessment methods: a randomized crossover feeding study.

Supplemental materials.

Supplemental Table 1: ACE-TADA controlled feeding study menu items

Week 1	Week 2	Week 3
	Breakfast	
Cornflakes	Cornflakes	Cornflakes
Weet-bix	Weet-bix	Weet-bix
Special K	Nutrigrain	Special K
Muesli, natural	Muesli, natural	Muesli, natural
Porridge	Porridge	Blueberries
Banana	Peaches	Yoghurt, plain, Greek
Peaches	Banana	Yoghurt, flavoured
Blueberries	Blueberries	Toast, fruit bread
Strawberries	Strawberries	Toast, ciabatta
Yoghurt, plain, Greek	Yoghurt, plain, Greek	Toast, multigrain or white
Yoghurt, flavoured	Yoghurt, flavoured	Jam or marmalade
Fruit bread	Toast, fruit bread	Honey
Toast, ciabatta	Toast, ciabatta	Peanut paste
Toast, multigrain or white	Toast, multigrain or white	Vegemite
Jam	Jam	Butter
Marmalade	Marmalade	Margarine
Honey	Honey	Avocado
Peanut paste	Peanut paste	Eggs, poached
Vegemite	Vegemite	Grilled tomato
Butter	Butter	Grilled mushroom
Margarine	Margarine	Dressing, balsamic
Avocado	Avocado	Toasted English muffin, cheese
Lemon	Lemon	Toasted English muffin, ham and cheese
Eggs, poached	Eggs, poached	Orange juice
Ham and cheese melts	Ham and cheese melts	Apple juice
Cheese melt	Cheese melt	Hot tea (tea bag)
Blueberry muffin	Water	Coffee (espresso, instant)
Water, still	Water, sparkling	Coffee, flat white
Water, sparkling	Orange juice	Choc milk
Orange juice	Apple juice	Iced coffee
Apple juice	Hot tea (tea bag)	Milk, 2% fat
Hot tea (tea bag)	Tea herbal	Milk, skim
Tea herbal	Coffee (espresso, instant)	Soy milk, regular
Coffee (espresso, instant)	Coffee, flat white	Soy milk, lite
Coffee, flat white	Choc milk	Sugar
Milk, 2% fat	Milk, 2% fat	
Milk, skim	Milk, skim	
Milk, regular	Milk, regular	
Soy milk, regular	Soy milk, regular	
Soy milk, lite	Soy milk, lite	

Milk, lactose-free	Milk, lactose-free	
Artificial sweetener	Artificial sweetener	
Sugar	Sugar	
Almond milk		

Lunch

Vegetarian sushi	Vegetarian sushi	Vegetarian sushi
Chicken sushi	Chicken sushi	Chicken sushi
Smoked salmon sushi	Smoked salmon sushi	Smoked salmon sushi
Chicken rice paper roll	Chicken rice paper roll	Chicken rice paper roll
Tofu rice paper roll	Tofu rice paper roll	Tofu rice paper roll
Pickled ginger	Pickled ginger	Pickled ginger
Wasabi	Wasabi	Wasabi
Soy sauce	Soy sauce	Soy sauce
Continental roll, chicken	Wrap, salad with chicken	Wrap, salad, cheese, avocado
Continental roll, ham	Wrap, to add cheese only	Wrap, salad, cheese, chicken
Toasted sandwich, ham, cheese & tomato	Wrap, salad with ham	Wrap, salad, cheese, ham
Toasted sandwich, cheese	Wrap, to add avocado only	Chicken salad, Thai style
Vietnamese salad, chicken	Wrap, salad, cheese, avocado	Fruit salad
Vietnamese salad, vegan, tofu	Toasted sandwich, ham, cheese & tomato	Chocolate brownie
Fruit salad	Toasted sandwich, cheese	Water, still
Choc chip cookie	Chicken and corn soup	Water, sparkling
Water, still	Greek salad	Orange juice
Water, sparkling	Dressing	Apple juice
Orange juice	Fruit salad	Diet coke
Apple juice	Carrot cake	Coke zero
Diet coke	Water, still	Coke
Coke zero	Water, sparkling	Ginger beer
Coke	Orange juice	Lemon squash
Ginger beer	Apple juice	Lemonade
Lemonade	Diet coke	Lemonade, diet
Lemonade, diet	Coke zero	Coffee (espresso, instant)
Lemon squash	Coke	Coffee, flat white
Coffee (espresso, instant)	Ginger beer	Hot tea (tea bag)
Coffee, flat white	Lemon squash	Milk, 2% fat
Hot tea (tea bag)	Lemonade	Milk, skim
Tea, herbal	Lemonade, diet	Soy milk, regular
Milk, 2% fat	Coffee (espresso, instant)	Soy milk, lite
Milk, skim	Coffee, flat white	Artificial sweetener
Milk, regular	Hot tea (tea bag)	Dressing
Soy milk, regular	Milk, 2% fat	Sugar
Soy milk, lite	Milk, skim	
Milk, lactose-free	Milk, regular	
Artificial sweetener	Soy milk, regular	
Sugar	Soy milk, lite	
Almond milk	Milk, lactose-free	
	Artificial sweetener	
	Sugar	
	Tea, herbal	

Dinner

Chicken satay with rice	Thai green curry	Chicken and vegetable curry and rice
Vegetarian lasagna	Rice	Cannelloni, spinach and ricotta
Beef lasagna	Cannelloni, spinach and ricotta	Spaghetti bolognese
Vegan rice	Tortellini, beef	Pizza, supreme
Teriyaki chicken noodles	Vegan fried rice	Roast chicken
Roast chicken	Vermicelli noodles with beef	Roast beef
Gravy	Roast chicken	Gravy
Steamed vegetables	Gravy	Garden salad
Baked potato	Quiche Lorraine	Greek salad
Green salad	Vegetable quiche	Dressing
Dressing	Green salad	Fruit salad
Fruit salad	Dressing	Ice cream stick, drumstick
Lions fruit cake	Steamed vegetables	Ice cream stick, Magnum
Ice cream stick, drumstick	Baked potato	Water, still
Ice cream stick, Magnum	Fruit salad	Water, sparkling
Choc chip cookie	Lions fruit cake	Orange juice
Water, still	Ice cream stick, drumstick	Apple juice
Water, sparkling	Ice cream stick, Magnum	Diet coke
Orange juice	Water, still	Coke zero
Apple juice	Water, sparkling	Coke
Diet coke	Orange juice	Ginger beer
Coke zero	Apple juice	Lemonade
Coke	Diet coke	Lemonade, diet
Ginger beer	Coke zero	Lemon squash
Lemonade	Coke	Cheddar cheese, grated
Lemonade, diet	Ginger beer	
Lemon squash	Lemonade	
	Lemonade, diet	
	Lemon squash	

Supplemental Table 2. Protocol for an image-assisted interviewer-administered 24hr dietary recall interview, conducted remotely via a video call. The interview structure is based on the Automated Multiple Pass Method (AMPM) developed by the USDA with adaptions outlined in the table.

AMPM	Image-assisted mFR24	Adaptions	When image not taken follow the AMPM script
**1. Quick List:**<br>Aim: The purpose of the QL pass is to get a quick report of foods and beverages consumed in the past 24 hours without interrupting the respondent and also to introduce the respondent to the concept of the 24 hour recall.	1. Quick List	Taken from the mini label list provided by the participant.	
**2. Forgotten foods and additions**<br>Aim: The FFL prompts the respondent's memory and collects other food or beverages the respondent did not report in the QL.	2. Forgotten foods and additions	After detail cycle, before final probe	Read out commonly forgotten foods list
**3. Time and Occasion**<br>Aim: The 'time and occasion' of food or beverages consumed are recorded.	3. Time and Occasion	Does not require a separate pass. Time of eating taken from the image metadata.	Ask participant to recall "time and occasion" of forgotten foods when item is reported
**4. Detail cycle**<br>Aim: to collect specific descriptive information about each food and beverage reported and also record quantities and any additions made to the foods.	4. Detail cycle	Clarify only non-identifiable food and beverage items<br><br>Follow the food model booklet to confirm amounts consumed<br><br>Check the after-image for leftovers	Follow the food model booklet to confirm amounts consumed
**5. Final Probe**<br>Aim: is the last opportunity for the respondent to remember any new foods and drinks	5. Final Probe		Read out the list of food and beverage items

Supplemental Table 3. STROBE-nut: An extension of the STROBE statement for nutritional epidemiology.

Lachat C et al. (2016) STrengthening the Reporting of OBservational studies in Epidemiology – Nutritional Epidemiology (STROBE-nut): an extension of the STROBE statement. Plos Medicine 13(6) http://dx.doi.org/10.1371/journal.pmed.1002036 pdf or online version.

Item	Item nr	STROBE recommendations	Extension for Nutritional Epidemiology studies (STROBE-nut)	Reported on page #
**Title and abstract**	1	(a) Indicate the study's design with a commonly used term in the title or the abstract.<br>(b) Provide in the abstract an informative and balanced summary of what was done and what was found.	**nut-1** State the dietary/nutritional assessment method(s) used in the title, abstract, or keywords.	1
**Introduction**				
Background rationale	2	Explain the scientific background and rationale for the investigation being reported.		6-10
Objectives	3	State specific objectives, including any pre-specified hypotheses.		10
**Methods**				
Study design	4	Present key elements of study design early in the paper.		12
Settings	5	Describe the setting, locations, and relevant dates, including periods of recruitment, exposure, follow-up, and data collection.	**nut-5** Describe any characteristics of the study settings that might affect the dietary intake or nutritional status of the participants, if applicable.	11-12

Item	Item nr	STROBE recommendations	Extension for Nutritional Epidemiology studies (STROBE-nut)	Reported on page #
Participants	6	a) Cohort study—Give the eligibility criteria, and the sources and methods of selection of participants. Describe methods of follow-up.<br><br>Case-control study—Give the eligibility criteria, and the sources and methods of case ascertainment and control selection. Give the rationale for the choice of cases and controls.<br><br>Cross-sectional study—Give the eligibility criteria, and the sources and methods of selection of participants.<br><br>(b) Cohort study—For matched studies, give matching criteria and number of exposed and unexposed.<br><br>Case-control study—For matched studies, give matching criteria and the number of controls per case.	**nut-6** Report particular dietary, physiological or nutritional characteristics that were considered when selecting the target population.	11-12
Variables	7	Clearly define all outcomes, exposures, predictors, potential confounders, and effect modifiers. Give diagnostic criteria, if applicable.	**nut-7.1** Clearly define foods, food groups, nutrients, or other food components.<br><br>**nut-7.2** When using dietary patterns or indices, describe the methods to obtain them and their nutritional properties.	23-24

Item	Item nr	STROBE recommendations	Extension for Nutritional Epidemiology studies (STROBE-nut)	Reported on page #
Data sources - measurements	8	For each variable of interest, give sources of data and details of methods of assessment (measurement).Describe comparability of assessment methods if there is more than one group.	**nut-8.1** Describe the dietary assessment method(s), e.g., portion size estimation, number of days and items recorded, how it was developed and administered, and how quality was assured. Report if and how supplement intake was assessed.<br><br>**nut-8.2** Describe and justify food composition data used. Explain the procedure to match food composition with consumption data. Describe the use of conversion factors, if applicable.<br><br>**nut-8.3** Describe the nutrient requirements, recommendations, or dietary guidelines and the evaluation approach used to compare intake with the dietary reference values, if applicable.<br><br>**nut-8.4** When using nutritional biomarkers, additionally use the STROBE Extension for Molecular Epidemiology (STROBE-ME). Report the type of biomarkers used and their usefulness as dietary exposure markers.<br><br>**nut-8.5** Describe the assessment of nondietary data (e.g., nutritional status and influencing factors) and timing of the assessment of these variables in relation to dietary assessment.	15-21

Item	Item nr	STROBE recommendations	Extension for Nutritional Epidemiology studies (STROBE-nut)	Reported on page #
			**nut-8.6** Report on the validity of the dietary or nutritional assessment methods and any internal or external validation used in the study, if applicable.	
Bias	9	Describe any efforts to address potential sources of bias.	**nut-9** Report how bias in dietary or nutritional assessment was addressed, e.g., misreporting, changes in habits as a result of being measured, or data imputation from other sources	22-23
Study Size	10	Explain how the study size was arrived at.		12
Quantitative variables	11	Explain how quantitative variables were handled in the analyses. If applicable, describe which groupings were chosen and why.	**nut-11** Explain categorization of dietary/nutritional data (e.g., use of N-tiles and handling of nonconsumers) and the choice of reference category, if applicable.	
Statistical Methods	12	(a) Describe all statistical methods, including those used to control for confounding<br>(b) Describe any methods used to examine subgroups and interactions.<br>(c) Explain how missing data were addressed.<br>(d) Cohort study—If applicable, explain how loss to follow-up was addressed.<br>Case-control study—If applicable, explain how matching of cases and controls was addressed.	**nut-12.1** Describe any statistical method used to combine dietary or nutritional data, if applicable.<br>**nut-12.2** Describe and justify the method for energy adjustments, intake modeling, and use of weighting factors, if applicable.<br>**nut-12.3** Report any adjustments for measurement error, i.e,. from a validity or calibration study.	22-24

Item	Item nr	STROBE recommendations	Extension for Nutritional Epidemiology studies (STROBE-nut)	Reported on page #
		Cross-sectional study—If applicable, describe analytical methods taking account of sampling strategy.<br><br>(e) Describe any sensitivity analyses.		
**Results**				
Participants	13	(a) Report the numbers of individuals at each stage of the study—e.g., numbers potentially eligible, examined for eligibility, confirmed eligible, included in the study, completing follow-up, and analyzed.<br><br>(b) Give reasons for non-participation at each stage.<br><br>(c) Consider use of a flow diagram.	**nut-13** Report the number of individuals excluded based on missing, incomplete or implausible dietary/nutritional data.	N/A
Descriptive data	14	(a) Give characteristics of study participants (e.g., demographic, clinical, social) and information on exposures and potential confounders<br><br>(b) Indicate the number of participants with missing data for each variable of interest<br><br>(c) Cohort study—Summarize follow-up time (e.g., average and total amount)	**nut-14** Give the distribution of participant characteristics across the exposure variables if applicable. Specify if food consumption of total population or consumers only were used to obtain results.	25

Item	Item nr	STROBE recommendations	Extension for Nutritional Epidemiology studies (STROBE-nut)	Reported on page #
Outcome data	15	Cohort study—Report numbers of outcome events or summary measures over time.<br><br>Case-control study—Report numbers in each exposure category, or summary measures of exposure.<br><br>Cross-sectional study—Report numbers of outcome events or summary measures.		27
Main results	16	(a) Give unadjusted estimates and, if applicable, confounder-adjusted estimates and their precision (e.g., 95% confidence interval).<br><br>Make clear which confounders were adjusted for and why they were included.<br><br>(b) Report category boundaries when continuous variables were categorized.<br><br>(c) If relevant, consider translating estimates of relative risk into absolute risk for a meaningful time period.	**nut-16** Specify if nutrient intakes are reported with or without inclusion of dietary supplement intake, if applicable.	N/A
Other analyses	17	Report other analyses done—e.g., analyses of subgroups and interactions and sensitivity analyses.	**nut-17** Report any sensitivity analysis (e.g., exclusion of misreporters or outliers) and data imputation, if applicable.	N/A
**Discussion**				

Item	Item nr	STROBE recommendations	Extension for Nutritional Epidemiology studies (STROBE-nut)	Reported on page #
Key results	18	Summarize key results with reference to study objectives.		35-36
Limitation	19	Discuss limitations of the study, taking into account sources of potential bias or imprecision. Discuss both direction and magnitude of any potential bias.	**nut-19** Describe the main limitations of the data sources and assessment methods used and implications for the interpretation of the findings.	40-41
Interpretation	20	Give a cautious overall interpretation of results considering objectives, limitations, multiplicity of analyses, results from similar studies, and other relevant evidence.	**nut-20** Report the nutritional relevance of the findings, given the complexity of diet or nutrition as an exposure.	36-39
Generalizability	21	Discuss the generalizability (external validity) of the study results.		40
**Other information**				
Funding	22	Give the source of funding and the role of the funders for the present study and, if applicable, for the original study on which the present article is based.		2
Ethics			**nut-22.1** Describe the procedure for consent and study approval from ethics committee(s).	11
Supplementary material			**nut-22.2** Provide data collection tools and data as online material or explain how they can be accessed.	16-21

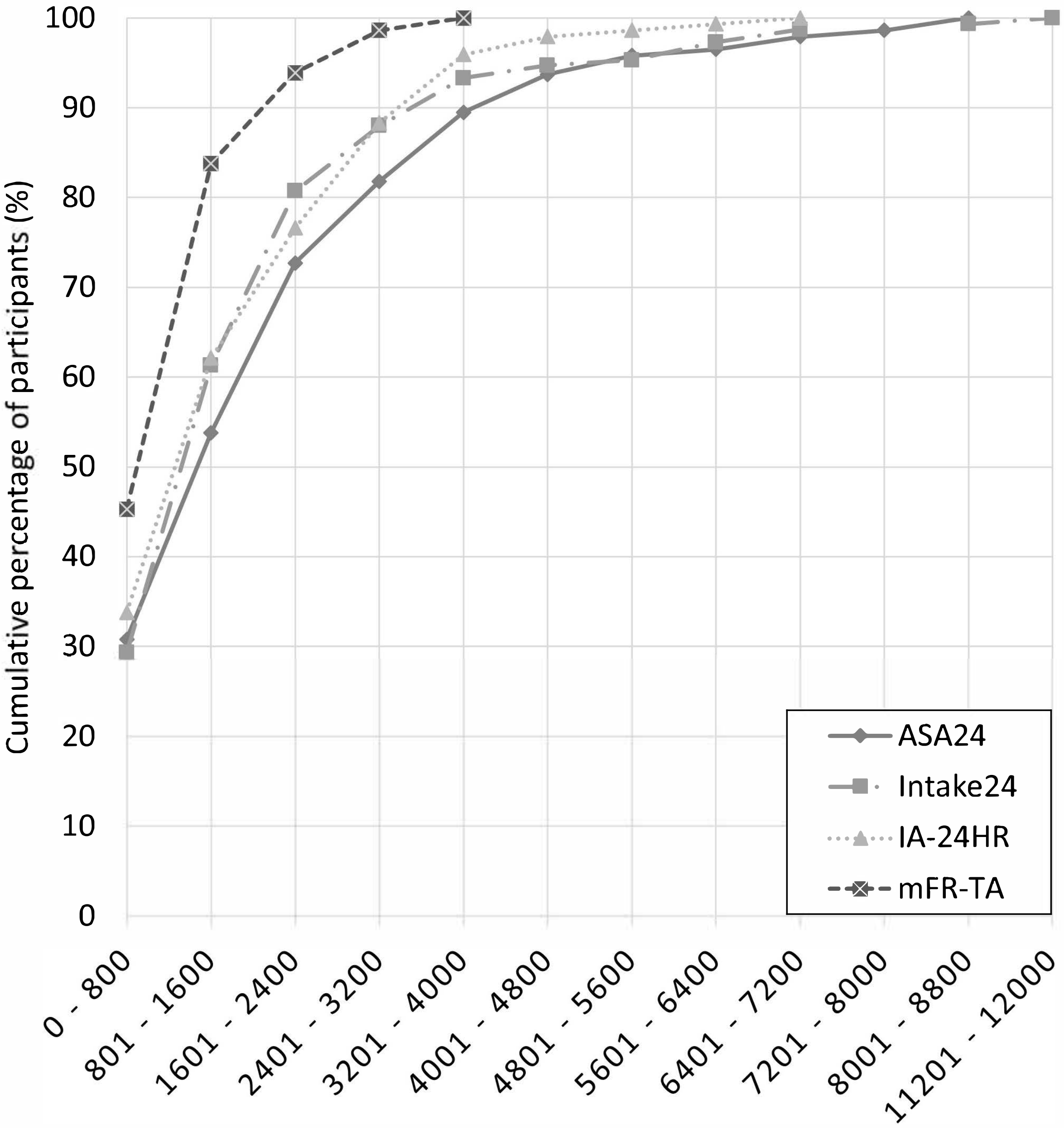

Supplemental figure 1. Cumulative percentage of participants within increments of energy intake estimation error, by method, showing absolute difference between observed and estimated energy intake (kJ).

Supplemental figure 2. Bland Altman scatterplots of percentage error in estimated carbohydrate intake against true carbohydrate intake, showing mean difference (solid lines). A, ASA24-Australia; B, Intake24-Australia; C, mFR-TA; D, IA-24HR.

Supplemental figure 3. Bland Altman scatterplots of percentage error in estimated protein intake against true protein intake, showing mean difference (solid lines). A, ASA24-Australia; B, Intake24-Australia; C, mFR-TA; D, IA-24HR.

Supplemental figure 4. Bland Altman scatterplots of percentage error in estimated total fat intake against true total fat intake, showing mean difference (solid lines). A, ASA24-Australia; B, Intake24-Australia; C, mFR-TA; D, IA-24HR.

Abbreviations:

ASA24 - Automated Self-Administered Dietary Assessment Tool-Australia

Intake24-Australia

mFR-TA - mobile Food Record – Trained Analyst

IA-24HR mage - Assisted Interviewer-Administered 24-hour recall

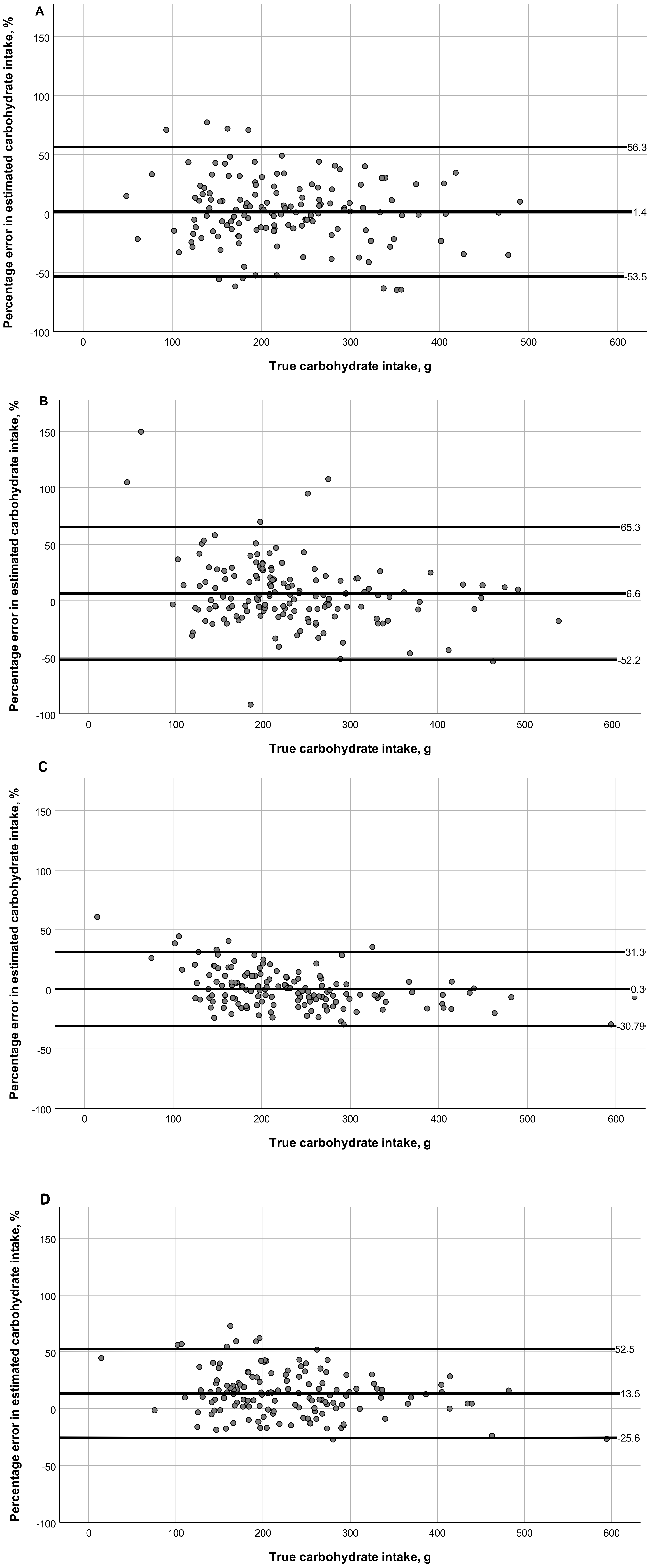

A
B
C
D
Percentage error in estimated carbohydrate intake, %
True carbohydrate intake, g
150
100
50
0
-50
-100
0
100
200
300
400
500
600
56.3
1.4
-53.5
65.3
6.6
-52.2
31.3
0.3
-30.79
52.5
13.5
-25.6

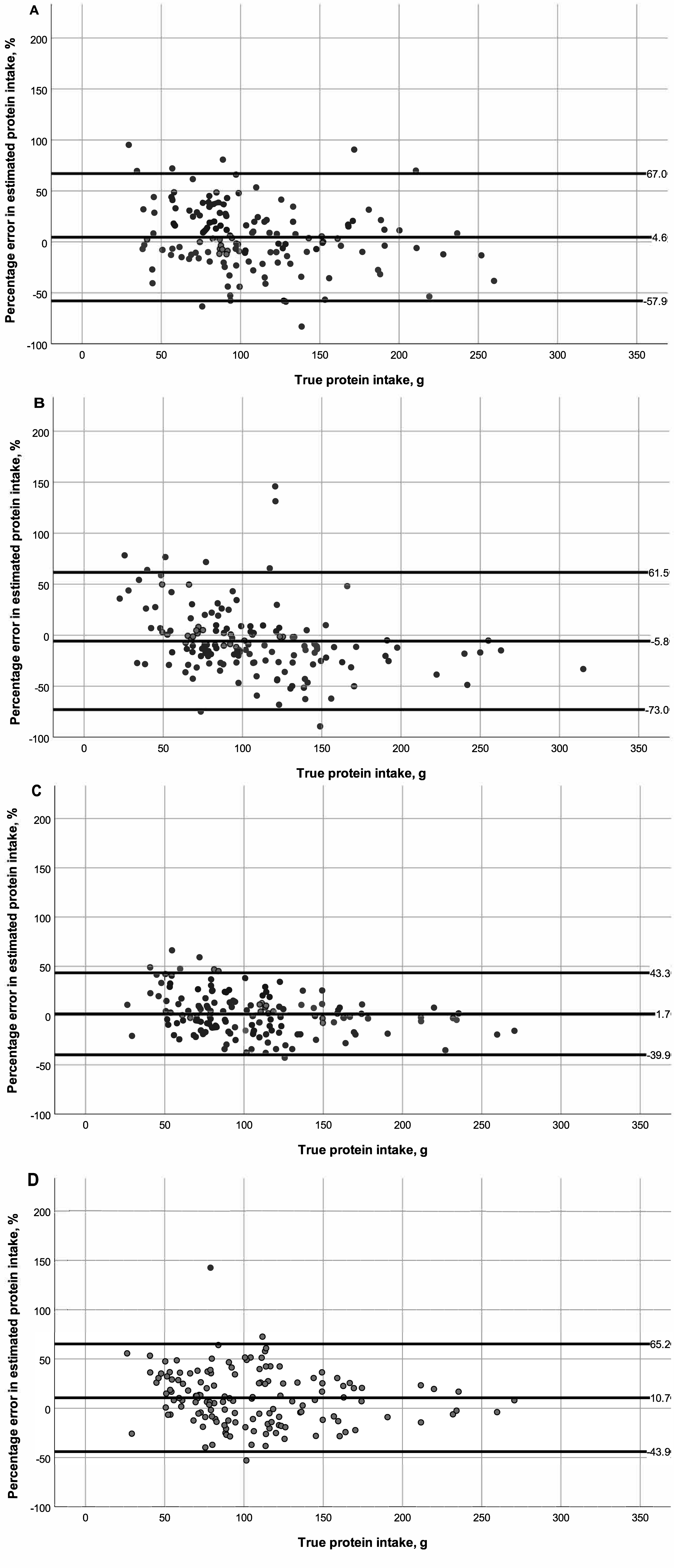
A
B
C
D
Percentage error in estimated protein intake, %
True protein intake, g
200
150
100
50
0
-50
-100
0
50
100
150
200
250
300
350
67.0
4.6
-57.9
61.5
-5.8
-73.0
43.3
1.7
-39.9
65.2
10.7
-43.9

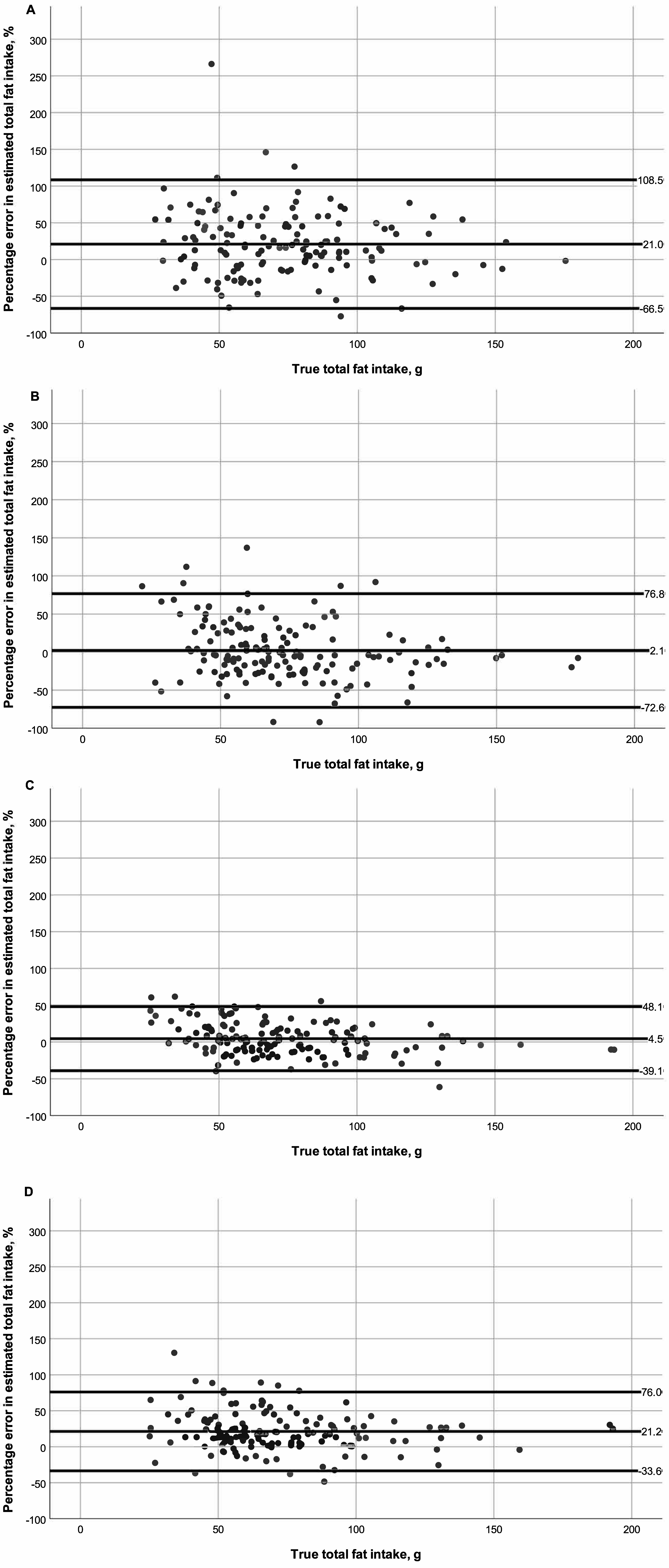
A
Percentage error in estimated total fat intake, %
True total fat intake, g
108.5
21.0
-66.5
B
Percentage error in estimated total fat intake, %
True total fat intake, g
76.8
2.1
-72.6
C
Percentage error in estimated total fat intake, %
True total fat intake, g
48.1
4.5
-39.1
D
Percentage error in estimated total fat intake, %
True total fat intake, g
76.0
21.2
-33.6
